# Identifying a Role of Red and White Wine Extracts in Counteracting Skin Aging: Effects of Antioxidants on Fibroblast Behavior

**DOI:** 10.3390/antiox10020227

**Published:** 2021-02-03

**Authors:** Sara Cruciani, Margherita Trenta, Giovanna Rassu, Giuseppe Garroni, Giacomo Luigi Petretto, Carlo Ventura, Margherita Maioli, Giorgio Pintore

**Affiliations:** 1 Department of Biomedical Sciences, University of Sassari, Viale San Pietro 43/B, 07100 Sassari, Italy; sara.cruciani@outlook.com (S.C.); giugarroni21@gmail.com (G.G.); 2 Department of Chemistry and Pharmacy, University of Sassari, Via F. Muroni 23/b, 07100 Sassari, Italy; margheritatrenta@virgilio.it (M.T.); grassu@uniss.it (G.R.); gpetretto@uniss.it (G.L.P.); pintore@uniss.it (G.P.); 3 Laboratory of Molecular Biology and Stem Cell Engineering-Eldor Lab, National Institute of Biostructures and Biosystems, Innovation Accelerator, CNR, Via Piero Gobetti 101, 40129 Bologna, Italy; ventura.vid@gmail.com; 4 Center for Developmental Biology and Reprogramming-CEDEBIOR, Department of Biomedical Sciences, University of Sassari, Viale San Pietro 43/B, 07100 Sassari, Italy

**Keywords:** cell proliferation, antioxidants, cell senescence, bioactive molecules, skin aging, oxidative stress

## Abstract

Dermal fibroblasts are the main actor in many proteins’ secretion, including collagen, preserving skin function. Free radicals are involved in skin aging and damages involving different cellular components. The imbalance between reactive oxygen species (ROS) amount and natural antioxidant enzymes negatively affects skin homeostasis. Natural compounds have recently emerged as a potential anti-aging tool in tissue regeneration. In the present paper we evaluated the antioxidant activity of white and red wines, considering their probable use, as raw materials, for the formulation of cosmetic products with anti-aging properties. We studied a method that would allow the removal of the alcoholic fraction of wines and determined their composition by LC-MS analysis. We then tested the possible cytotoxic effects of red and white wines on fibroblasts by 3-(4,5-dimethylthiazol-2-yl)-2,5-diphenyltetrazolium (MTT) assay, and their antioxidant activity by the catalase activity test in stressing conditions. Finally, we evaluated their anti-aging potential through the β-galactosidase colorimetric assay. Our results showed that wine extracts exhibit a remarkable antioxidant and anti-aging activity, especially on cells exposed to a marked stressful event. These properties could suggest their possible application as cosmetical products for skin regeneration.

## 1. Introduction

The skin is the most extensive organ of the body and has a multiplicity of functions, protecting the underlying tissues from chemical and mechanical insults, UV radiation, free radicals, and infections. It plays a role in thermoregulation, has endocrine and biochemical functions, and is the organ of application and/or absorption of xenobiotics (drugs, poisons, cosmetics) [1,2,3]. In the dermis, elastin fibers and collagen fibers ensure the right skin elasticity. Age, hormones, and the damaging effects of ultraviolet radiation can reduce the thickness and elasticity of the dermis, resulting in wrinkles and loss of skin tone [4,5]. It is already known that skin aging compromises the barrier function of the skin, resulting in a dry appearance and susceptibility to environmental aggressors [6]. The reactive oxygen species (ROS), resulting from the loss of electrons during aerobic metabolism or after exposure to environmental factors, are unstable species able to damage different biomolecules [7,8]. To counter their effect, the natural antioxidant defenses in the body are able to keep ROS within physiologically acceptable levels. It seems, in fact, that ROS, if present in controlled amounts, also have a physiological action, functioning as signal molecules between cells [9,10]. This imbalance between ROS and antioxidant enzymes, such as catalase, glutathione reductase, and superoxide dismutase, causes damages to proteins, lipids, and DNA [11], thus negatively interfering with the intracellular signaling pathways of keratinocytes and fibroblasts and altering the expression of MMP (matrix metalloproteinases), procollagen, and pro-inflammatory cytokines [12,13]. Phenolic compounds as resveratrol, hydroxytyrosol, and epigallocatechin-3-gallate, present in vegetables, fruits, and derivatives, are the main defense molecules against fungi, bacteria, and virus [14]. The beneficial effects of polyphenols—widely present in wine—have attracted considerable attention in the pharmaceutical and cosmetic industry in recent years [15,16]. Polyphenol consumption may play protective effects in acute and chronic diseases, in the regulation of metabolism and cell proliferation [17]. The development of a single efficient procedure for the extraction and characterization of phenolic compounds has many limitations, mainly due to the structural diversity of phenolic compounds and their interaction with other cellular components [18,19]. Modern green extraction techniques and high-resolution mass spectrometry (LC-ESI-LTQ-Orbitrap-MS) represent promising approaches for the management of these bioactive molecules [20]. Therefore, we studied a cheap, flexible, robust, and efficient method that would allow the removal of the alcoholic fraction of wines, usable as cosmetic products. The numerous health effects linked to the consumption of wine have been known for a long time, and in particular the high content of antioxidant polyphenolic compounds makes it useful in the treatment of diseases with high oxidative stress [21,22]. Topical application of antioxidants can support the skin’s antioxidant system against oxidative stress and can protect it from long-term photo-aging [23,24]. Within this context, in the present paper we aimed at evaluating the antioxidant effects of red and white wine extracts in cells exposed to a strong stressful event, in order to counteract cell senescence, suggesting their possible inclusion in different topical formulations with anti-aging properties, for the treatment of mature and damaged skin.

## 2. Materials and Methods

The red and white wines used in this study were the Buio, with Denomination of Controlled Origin, obtained from Carignano del Sulcis grapes, produced by Cantina Mesa, Sardegna, and Giunco, produced by the same Cantina Mesa, obtained from Vermentino grapes.

For in vitro experiments, in-line human skin fibroblasts (HFF1) (ATCC, Manassas, VA, USA) at passage 5 were used. The cells were grown in the presence of a basal growth medium, composed of Dulbecco’s modified Eagle’s medium (DMEM) (Life Technologies Grand Island, NY, USA) supplemented with 10% fetal bovine serum (FBS) (Life Technologies, Grand Island, NY, USA), 200 mM L-glutamine (Euroclone, Milano, Italy), and 200 U/mL penicillin—0.1 mg/mL streptomycin (Euroclone, Milano, Italy). The cells were grown in thermostatic incubators at 37 °C and 5% (*v/v*) CO_2_.

### 2.1. Preparation of Wine Extracts: Spray Dryer

During the initial steps of research, we studied a method for removing the alcoholic fraction of wines, toxic for the cells. A mini Spray Dryer B-191 (BUCHI Italia s.r.l, Cornaredo, Italy) was used for the drying procedure. Initially, an aliquot of 100 mL of wines was dried with inlet and outlet temperatures (nitrogen), respectively, of 25 and 70 °C; the flow rate was set to 15%. Subsequently, a 100 mL portion of the wine was added with 0.2 g of xanthan gum and a minimum volume of water necessary for the solubilization of the gum. The inlet and outlet temperatures (nitrogen) were 135 and 70 °C, respectively; the feed flow rate was set to 12%.

### 2.2. Preparation of Wine Extracts: Rotavapor

We evaporated 500 g exactly weighed red and white wines by Buchi Rotavapor^®^ R-110 (BUCHI Italia s.r.l, Cornaredo, Italy) in 500 mL flasks, with a temperature of 55 °C, flask rotation speed equal to 5, and vacuum conditions equal to 60 mmHg. Initially, the 2 wine samples were brought completely dry. Then, we subjected them to controlled evaporation, with a defined time of 20 min, to remove the alcoholic fraction.

### 2.3. HPLC Analysis

Liquid chromatography–mass spectrometry (LC-MS) analysis was carried out according to D’Urso et al. (2020) [25] with slight modifications. Briefly, 5 µL of red and white wines before and after rotavapor evaporation at the final concentration of 1 mg/mL (in H_2_O) were injected in a liquid chromatography system constituted of a quaternary Accela 600 pump and an Accela autosampler, connected to a linear Trap-Orbitraphybrid mass spectrometer (LTQ-Orbitrap XL, Thermo Fisher Scientific, Bremen, Germany) with electrospray ionization (ESI). Chromatographic separation was conducted on a C18 Moon column (100 × 2.0 mm, particle size 5µm; Phenomenex), using 0.1% formic acid (solvent A) and 0.1% formic acid (solvent B) H_2_O and CH_3_CN as eluent phases. The following binary gradient was applied at 200 µL/min: 0–35 min, 5 to 95% (B) and 35–40 min, isocratic 95% (B ESI source parameters were as follows: Capillary voltage −48 V; tube lens voltage −176.47; capillary temperature 280 °C; Sheath and Auxiliary Gas flow (N2), 15 and 5; Sweep gas 0; Spray voltage 5. MS spectra were acquired by full range acquisition covering m/z 180–1400. For fragmentation studies, a data-dependent scan experiment was performed, selecting precursor ions corresponding to most intensive peaks in LC–MS analysis. Xcalibur software version 2.1 was used for instrument control, data acquisition, and data analysis.

### 2.4. MTT Viability Assay

To evaluate their possible cytotoxic effect, the red and white wine extracts were tested on HFF1 at different concentrations (100, 200, 300, 400, and 500 mg/mL) from 24 to 72 h total, using the colorimetric test of 3-(4,5-dimethylthiazol-2-yl)-2,5-diphenyltetrazolium (MTT) (Sigma-Aldrich, Saint Louis, MO, USA). Vital cells were able to reduce this compound, producing formazan that can be quantified by spectrophotometer at 570 nm. The HFF1 were seeded at a concentration of 5000 cells/well in 96-well plates. Cells used as untreated control were grown in the only basic growing medium. At the end of the incubation period, the medium containing the extracts was removed, and 100 μL of MTT at the final concentration of 0.65 mg/mL was added to each well and incubated for 2 h. After incubation, the formazan was dissolved in DMSO and the absorbance was detected by spectrophotometric reading at 570 nm (Akribis Scientific, Common Farm, Frog Ln, Knutsford WA16 0JG, Great Britain). The viability of cultured cells in the presence of the various extracts was calculated as % cell viability compared to the untreated control:

(OD570 treated cells) × 100/ (OD570 control).
(1)

### 2.5. Antioxidant Activity

The antioxidant activity of wine extracts was evaluated by testing the catalase activity, an enzyme capable of degrading hydrogen peroxide in water and oxygen. The colorimetric assay used (Catalase Assay Kit) (Sigma-Aldrich, Saint Louis, MO, USA) allows evaluating the activity of this enzyme in treated cells by spectrophotometric reading. Cells were induced to senescence by 1 h treatment with 100 μM hydrogen peroxide (H_2_O_2_) and subsequently cultivated in the presence of the extracts in various concentrations (100, 200, 300, 400, and 500 mg/mL) for 24, 48, and 72 h. The cells used as a positive control of oxidative stress were grown in the basal growing medium following exposure to H_2_O_2_. Cells used as control were grown in the basal growing medium alone, without pre-exposure to H_2_O_2._ At the end of the incubation time, the samples, both treated and controls, were incubated with the reagents present in the kit at room temperature for 15 min to evaluate the development of color, and the absorbance of each was measured by spectrophotometric reading at 520 nm (Akribis Scientific, Common Farm, Frog Ln, Knutsford WA16 0JG, Great Britain). The activity of catalase was calculated on the number of micromoles present in each sample and compared with the activity of the untreated control.

### 2.6. β-Galactosidase Senescence Assay

The β-galactosidase colorimetric assay (Cell Signaling, MA, USA) was used to identify senescent cells in culture. HFF1 were grown in 24-well plates, in the presence of white and red wine extracts, at a concentration of 500 mg/mL, for a total of 72 h. The cells were previously induced to senescence by 1 h treatment with 100 μM hydrogen peroxide (H_2_O_2_). At the end of the incubation time, the medium containing the peroxides was removed, and the fresh medium containing conditioned concentrates was added to the cells. Cells used as an untreated control were cultured in the presence of the growing medium alone, without prior exposure to H_2_O_2_. Instead, cells pre-treated with peroxides were used as a positive control of senescence, cultured in the normal growing medium. After 72 h of treatment with the extracts, all cells were fixed and incubated with the dye for observation under the light microscope.

### 2.7. Statistical Analysis

Statistical analysis was performed using Statistical Package for the Social Sciences version 13 Software (SPSS Inc., Chicago, IL, USA). The experiments were performed 2 times with 3 technical replicates for each treatment. The distributions of each group variance were evaluated with the Kruskal–Wallis rank sum and Wilcoxon signed-rank test, assuming a *p*-value < 0.05 as statistically significant.

## 3. Results

### 3.1. Wine Extraction by Rotavapor Improves Quality of Extraction Maintaining the Phenolic Profiles of Wines

The first attempt to obtain the wine extract was carried out by a spry dryer, which was a fast and simple technique suitable for removing the alcoholic fraction from wines. Anyway, the obtained extract was not suitable for our scope, therefore, a simple and cheaper evaporation under vacuum was considered to obtain the sample for the biological and chemical analysis. Starting from 500 g of wine and working at 55 °C, after 20 min, 262 g of extract were recovered.

**Figure 1:**
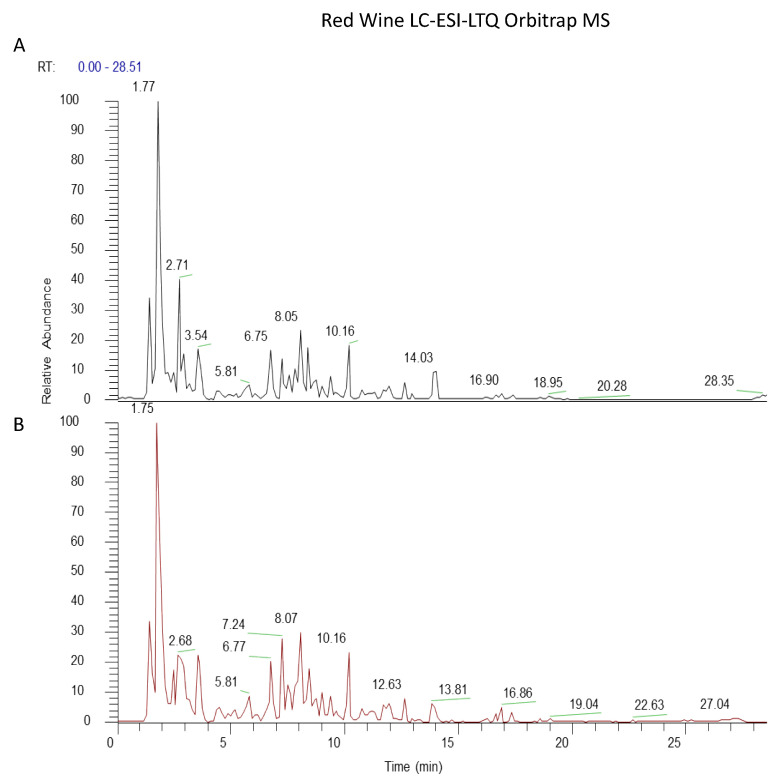
Chemical LC-ESI-LTQ-Orbitrap mass spectrometry (MS). profiles of red wine samples before (**A**) and after evaporation (**B**).

**Figure 2:**
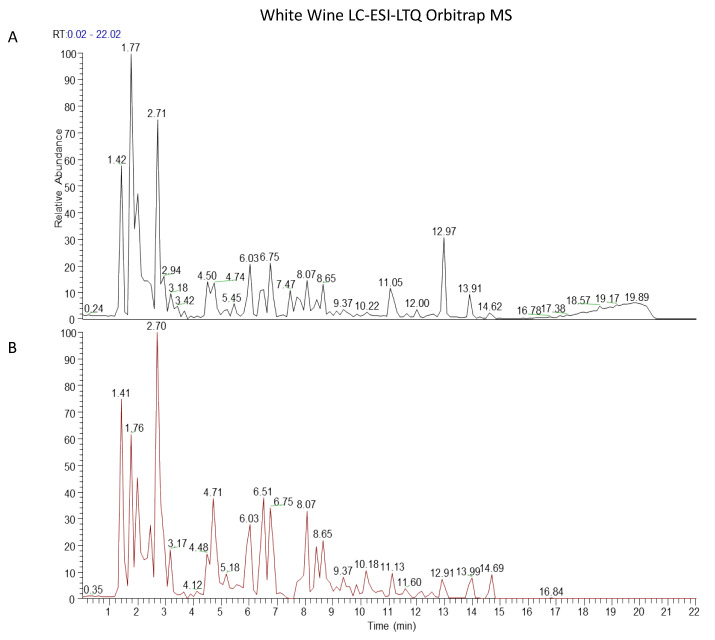
Chemical LC-ESI-LTQ-Orbitrap MS profiles of white wine samples before (**A**) and after evaporation (**B**).

In order to detect any deterioration of the polyphenols contained in the wine during the evaporation step, the samples before and after the treatment were subjected to the liquid chromatography analysis. Figure 1 and Figure 2 show the LC-ESI-LTQ-Orbitrap MS profiles of red and white wines before and after evaporation. The data acquisition was carried out in negative ionization mode; it was known that the negative ionization mode was more selective and allows to obtain greater sensitivity for phenolic compounds. 33 phenolic compounds have been identified in the red wine samples, and 26 compounds in the white wine samples (Table 1 and Table 2). The fingerprint showed the presence of phenolic acids, catechins and related proanthocyanidins, stilbenes, and flavonoid glycosides in the samples. The profiles of red wine before and after evaporation revealed slight quantitative differences (Figure 1). As expected, the red wine chromatograms were more crowded if compared to those of white wine, meaning that red wine contained more polyphenol compounds than the white one. (Figure 2). From a qualitative point of view, the raw samples and the correspondent wine extract were equivalent.

### 3.2. Wine Extracts Improve Cell Viability and Antioxidant Response

**Figure 3:**
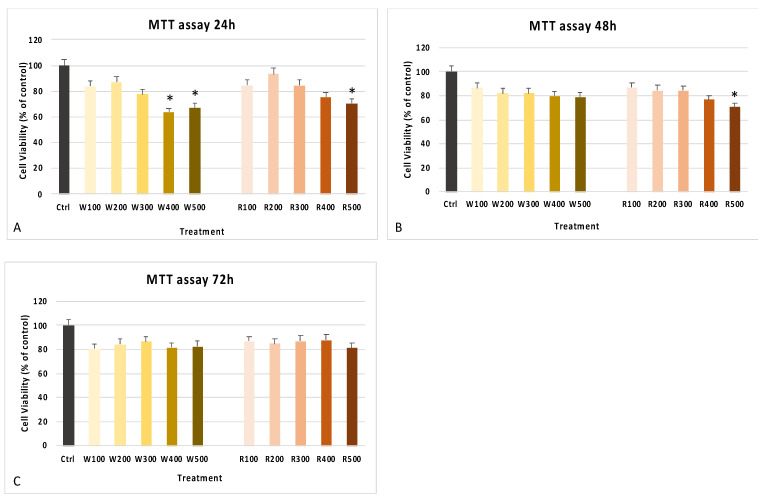
MTT vitality assay. The cytotoxicity of the extracts was evaluated in cultured cells in the presence of white and red wine extracts at concentrations of 100, 200, 300, 400, and 500 mg/mL for 24 h (**A**), 48 h (**B**), and 72 h (**C**). Cell viability was compared to untreated controls, cultured in the presence of the only basic growing medium, and expressed as absorbance at 570 nm. The experiments were performed two times with three technical replicates for each treatment. Data are expressed as mean ± SD referring to the control (∗ *p* ≤ 0.05) and evaluated with Kruskal–Wallis rank sum and Wilcoxon signed-rank tests.

**Figure 4:**
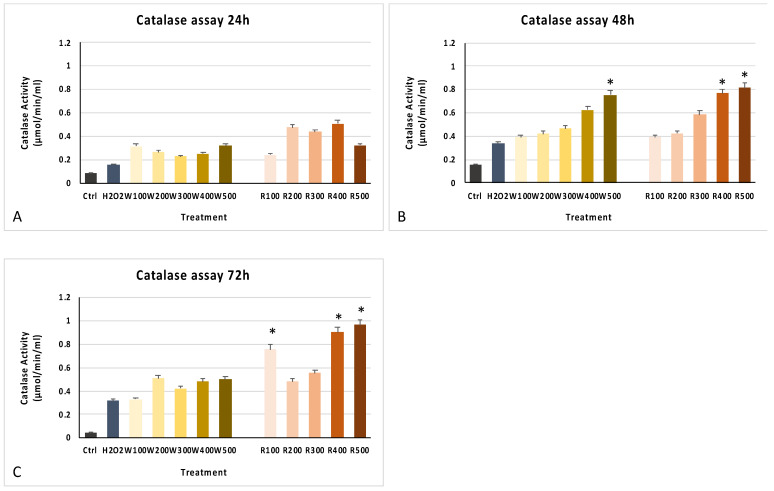
Catalase activity. The activity of catalase was evaluated in cells pretreated with H_2_O_2_ and grown in the presence of white and red wine extracts at concentrations of 100, 200, 300, 400, and 500 mg/mL for 24 h (**A**), 48 h (**B**), and 72 h (**C**). The activity of wine extracts-treated cells was compared to H_2_O_2_-untreated controls, cultured in the presence of the only basic growing medium (Ctrl). Positive control of oxidative stress were cells pre-treated with H_2_O_2_ alone (H_2_O_2_). The absorbance of the various samples was measured at 520 nm. The experiments were performed two times with three technical replicates for each treatment. Data are expressed as mean± SD referring to the control (∗ *p* ≤ 0.05) and evaluated with Kruskal–Wallis rank sum and Wilcoxon signed-rank tests.

The MTT assay showed the non-toxicity of the wine extracts for all tested concentrations (Figure 3) and time of exposure, maintaining cell viability compared to control untreated cells. Only for higher concentrations (400 and 500 mg/mL of white and 500 mg/mL of red wine extracts), cells showed a significantly decreased cell viability after 24 h (panel A) and 48 h (panel B), as compared to control untreated cells. Cells treated with the various extracts also showed better antioxidant activity, stimulating catalase activity in the degradation of H_2_O_2_ in oxygen and water, protecting cells from oxidative stress-induced damages (Figure 4). The improvement of antioxidant activity in treated cells, as compared to controls, was already visible after 24 h of treatment, especially for higher concentrations (500 mg/mL) (Figure 4A), reaching a peak at 48 h, statistically significant for higher concentrations (Figure 4B) and then stabilized after 72 h (Figure 4C).

### 3.3. The Wine Extracts Counteract Cellular Aging, Despite Exposure to a Strong Stressful Event

**Figure 5:**
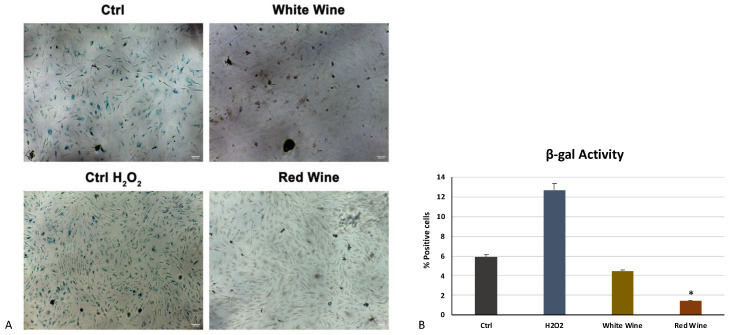
β-galactosidase activity. (**A**) The activity of β-galactosidase was evaluated in cells pretreated with H_2_O_2_ and grown in the presence of white and red wine extracts at a concentration of 500 mg/mL. Scale bar = 100 μm. (**B**) Senescent (blue) cells were evaluated by light microscopic observation. The percentage of SA-β-Gal-positive cells for each treatment was calculated as the number of positive cells divided by the total number of cells counted using an image software analysis (ImageJ). Data are expressed as mean ± SD referring to the control (∗ *p* ≤ 0.05).

Figure 5 shows the activity of β-galactosidase under various growing conditions. Cells cultured in the presence of the two extracts (white and red at 500 mg/mL) showed a clear reduction in the number of blue positive cells, and, therefore, senescent, as compared to untreated control cells cultured in the presence of the only growing medium (Ctrl) and to cells exposed to H_2_O_2_ without extracts (Ctrl H_2_O_2_).

## 4. Discussion

Polyphenols are the most abundant bioactive molecules in wine, also recently focusing of interest in the field of cosmetic applications [26]. Polyphenols are a group of compounds widely found in plants, very different from a structural point of view but responsible for the organoleptic and nutritional properties of foods and plants [14]. They also have a positive effect, protecting against cancer, cardiovascular diseases, diabetes, osteoporosis, and neurodegenerative diseases [27,28,29]. Other authors previously described their properties to counteract oxidative stress and inflammation [30]. In particular, the well-known preventive effect of atherosclerosis depends on the antioxidant activity on LDL cholesterol, whose oxidation would lead to the capture by white blood cells followed by the formation of atheromatous plaque [31,32]. Within this context, wine recently emerged as one of the best ways to prevent intestinal infection, showing antiviral and anti-bacterial activity against Gram-positive and Gram-negative microorganisms, as salmonellosis, shigellosis, colibacillosis, staphylococci, and streptococci [33,34].

Resveratrol is considered one of the most effective antioxidants present in wine, protecting skin from free radicals and delaying the aging process by the inhibition of tyrosinase activation [35]. Moreover, it influences the production of glycosaminoglycans, which facilitate and regulate the redistribution of water in the dermis, restoring its balance and leading to lasting hydration [36]. In addition, resveratrol modulates cell cycles, apoptosis, and senescence, showing protective effects against DNA oxidative damage [37]. Gallic acid and all its derivatives are considered the most important phenolic acids, with a high free radical scavenging activity, able to interfere with cell signaling pathways and apoptosis of cancer cells [38]. Flavonoids and anthocyanins exert a significant antioxidant activity. It is well known for their marked ability to reduce cancer cell proliferation and to protect from cardiovascular disease, obesity, and diabetes [39,40]. In addition, they are also involved in modulating neuronal functions, preventing age-related diseases [41]. An efficient extraction procedure is of critical importance in order to maintain the stability of phenolic compounds [42]. Within this context, in the present paper, we evaluated the components of two types of wine extracts obtained under vacuum evaporation to produce an alcohol-free concentrate. To be sure that the evaporation process did not affect the quality and quantity of the wine, the chemical profiles of the red and white wine samples were analyzed and compared with those after evaporation. The fingerprint analysis of samples showed the presence of large amounts of phenolic acids, catechins and related proanthocyanidins, stilbenes, and flavonoids glycosides (Figure 1 and Figure 2), useful as a raw material for the topical cosmetical preparation [43]. Their potential therapeutic effect in many skin disorders, including infected wounds and UV-irradiation, is probably related to the synergistic action of these bioactive molecules [44]. These compounds can inhibit ROS-generating enzymes such as xanthine oxidase and nicotinamide adenine dinucleotide phosphate (NADPH) oxidase [45,46].

Oxidative stress is caused by an excess of reactive oxygen species. ROS, derived from the loss of electrons during aerobic metabolism or after exposure to environmental factors, are unstable species able to induce changes in biomolecules structure and function [47,48]. Natural antioxidant defenses are involved in keeping ROS within physiologically suitable levels [49,50]. Any changes in this process can negatively affect skin aging [51,52]. Within this context, many natural extracts are well known for their beneficial effects in stimulating wound healing and antioxidant responses in damaged skin after exposure to environmental stress [53,54,55,56]. It is now known that skin aging leads to the impairment of barrier function, resulting in a dry appearance and susceptibility to environmental aggressors, therefore, representing a higher risk for skin disorders [57,58]. Moreover, wound healing is a complex and dynamic process of repair, restoring skin integrity, and tissue homeostasis [59]. Topical application of active antioxidant molecules can support the skin antioxidant system against oxidative stress, thus protecting it from long-term photo-aging [24,60]. It is well known that bioactive molecules, acting as antioxidants, are able to counteract the cell senescence and aging processes. Cell senescence is a stable phase of cell growth arrest featured by the secretion of secretory factors associated with senescence phenotypes (SASP) [61]. SASP can modulate neighboring cells, leading to the activation of signaling cascades involved in different pathological processes [62]. Senescent cells are associated with telomeres shortening and a steady pro-inflammatory milieu, promoting cell transdifferentiation [63]. Within this context, we have recently demonstrated that *Myrtus Communis L.* shows important antioxidant and regenerative properties by modulating stem cell pluripotency and inflammatory response [64]. Extracts from this plant, rich in polyphenols, are able to protect cells from oxidative stress, inducing the expression of telomerase reverse transcriptase (TERT) and counteracting premature senescence [65].

Other authors previously showed that therapeutic interventions towards senescent cells could restore health by counteracting chronic inflammation, inducing wound repair [66]. Nevertheless, other authors also revealed that quercetin, flavonoids, and gallic acid can prevent injuries caused through the direct free radical scavenging activity, supporting cell detoxification systems, such as superoxide dismutase, catalase, and glutathione peroxidase [67]. Catalase is one of the most important enzymes involved in ROS detoxification, whose dysregulation leads to many age-associated degenerative diseases [68,69]. It is well known that a catalase deficiency is related to an accelerated diabetic renal injury through peroxisomal dysfunction [70], thus influencing biological processes, including cell proliferation, differentiation, migration, and apoptosis [71]. In the present paper, we show that red and white wine extracts, at different concentrations, are able to counteract cell senescence induced by oxidative stress, modulating the activity of catalase, the main enzymes involved in the regulation of oxidative stress (Figure 4) and β-galactosidase (Figure 5). Our results demonstrate that wine extracts are able to counteract ROS accumulation, increasing catalase activity after H_2_O_2_ treatment, thus preventing chronic skin diseases and reducing the number of senescent cells. Taken together, our findings suggest that these wine extracts, obtained under vacuum evaporation, may thus represent an interesting raw material for the formulation of new cosmetic products to counteract skin aging. Further in vitro and in vivo studies also on single compounds may be helpful to translate these results in future applications for tissue regeneration.

## 5. Conclusions

Skin aging is a dynamic and multifactorial process induced by UV exposure and the related formation of reactive oxygen species. The only known defenses against photo-aging are sun filters and antioxidants, especially in combination, to reduce and neutralize free radical production. In the present paper, we focused the attention on the antioxidant potential of wine extracts, whose flavonoids are able to counteract aging, especially when applied on cells exposed to a marked stressful event.

Thanks to a novel extraction procedure, we removed the alcoholic fraction without qualitatively altering the flavonoid antioxidant components. From an economic point of view, it is clear that wine is more expensive than several by-products. It is well known that the cosmetic industry also uses several raw materials more expensive than wine. Red and white wine extracts may thus represent an interesting raw material for the formulation of new cosmetic products to counteract skin aging improving tissue regeneration.

## Figures and Tables

**Table 1:** LC-ESI-LTQ-Orbitrap MS and LC-ESI-LTQ-Orbitrap MS/MS identification of phenolic compounds in the red wine sample. The mass value was compared with the exact mass value calculated through ppm calculation, and the fragmentations obtained through LC-MS/MS experiments in the Dependent Ion Scan mode.

	tR (min)	Compound	Formula	Precursor Ion [M−H]−*m*/*z*	Dppm	Fragments [M−H]−*m*/*z*
1	4.49	Gallic acid	C_7_H_6_O_5_	169.0141	0.93	125
2	4.96	Procyanidin trimer	C_45_H_38_O_18_	865.1975	0.08	847/739/695/577
3	5.81	Protocatechuic acid	C_7_H_6_O_4_	153.0187	4.5	109
4	6.42	Quercetin-3-*O*-galactoside	C_21_H_20_O_12_	463.0863	−1.797	301
5	7.24	Procyanidin B1	C_30_H_26_O_12_	577.1339	−0.352	451/425/289
6	7.47	Caftaric acid	C_13_H_12_O_9_	311.0405	2.481	179
7	7.50	*p*-Coumaric acid	C_9_H_8_O_3_	163.0399	5.016	119
8	8.29	Procyanidin trimer	C_45_H_38_O_18_	865.1975	−0.694	847/739/695/577
9	8.41	Catechin	C_15_H_14_O_6_	289.0711	1.541	245/205
10	8.65	Fertaric acid	C_14_H_14_O_9_	325.0562	2.374	193
11	8.65	Vanillic acid	C_8_H_8_O_4_	167.0347	4.758	152/123
12	8.76	Procyanidin B2	C_30_H_26_O_12_	577.1345	1.018	451/425/289
13	9.01	Coutaric acid	C_13_H_12_O_8_	295.0454	2.021	163
14	9.35	Epicatechin	C_15_H_14_O_6_	289.0711	1.541	245/205
15	9.37	Ferulic acid	C_10_H_10_O_4_	193.0503	4.065	178/149
16	9.47	Rutin	C_27_H_30_O_16_	609.1453	0.409	301
17	9.59	Caffeic acid	C_9_H_8_O_4_	179.0347	4.349	135
18	10.05	Myricetin-3-*O*-glucoside	C_21_H_20_O_13_	479.0819	−0.023	317
19	10.27	Myricetin-3-*O*-glucuronide	C_21_H_18_O_14_	493.0612	−0.064	317
20	11.00	Kaempferol-3-*O*-galactoside	C_21_H_20_O_11_	447.0921	−0.263	285
21	11.09	Quercetin-3-*O*-glucoside	C_21_H_20_O_12_	463.0874	0.578	301
22	11.09	Quercetin-3-*O*-Rhamnoside	C_21_H_20_O_11_	447.0921	−0.263	301
23	11.31	Syringic acid	C_9_H_10_O_5_	197.0453	4.413	182/153
24	11.32	*trans-*Resveratrol glucoside	C_20_H_22_O_8_	389.1233	0.427	227
25	11.35	Quercetin glucuronide	C_21_H_18_O_13_	477.0665	0.279	301
26	11.39	Ethyl gallate	C_9_H_10_O_5_	197.0453	0.87	169
27	11.67	astilbin	C_21_H_22_O_11_	449.1080	0.428	303
28	12.27	Kaempferol-3-*O*-glucoside	C_21_H_20_O_11_	447.0921	−0.263	285
29	12.63	*cis*-Resveratrol glucoside	C_20_H_22_O_8_	389.1233	0.427	227
30	12.73	*trans* Resveratrol	C_14_H_12_O_3_	227.0710	0.679	185
32	16.50	*cis*-Resveratrol	C_14_H_12_O_3_	227.0706	0.319	185
33	16.86	dimetilcaffeic acid	C_11_H_12_O_4_	207.0660	0.795	179

**Table 2:** LC-ESI-LTQ-Orbitrap MS and LC-ESI-LTQ-Orbitrap MS/MS identification of phenolic compounds in the white wine sample. The mass value was compared with the exact mass value calculated through ppm calculation, and the fragmentations obtained through LC-MS/MS experiments in the Dependent Ion Scan mode.

	tR (min)	Compound	Formula	Precursor Ion [M−H]−*m*/*z*	Dppm	Fragments [M−H]−*m*/*z*
1	4.48	Gallic acid	C_7_H_6_O_5_	169.0135	891	125
2	4.93	Procyanidin trimer	C_45_H_38_O_18_	865.1984	0.855	847/739/695/577
3	6.98	Protocatechuic acid	C_7_H_6_O_4_	153.0192	5371	109
4	7.22	Procyanidin B1	C_30_H_26_O_12_	577.1340	−0.039	451/425/289
5	7.59	Caftaric acid	C_13_H_12_O_9_	311.0403	1.581	179
6	8.41	Procyanidin trimer 4	C_45_H_38_O_18_	865.1984	0.855	847/739/695/577
7	8.41	Catechin	C_15_H_14_O_6_	289.0710	1.299	245/205
8	8.77	Procyanidin B2	C_30_H_26_O_12_	577.1340	−0.039	451/425/289
9	8.77	Coutaric acid	C_13_H_12_O_8_	295.0451	0.767	163
10	9.49	Rutin	C_27_H_30_O_16_	609.1451	−0.593	301
11	9.37	Epicatechin	C_15_H_14_O_6_	289.0710	1299	245/205
12	9.37	Ferulic acid	C_10_H_10_O_4_	193.0505	5204	178/149
13	9.37	Fertaric acid	C_14_H_14_O_9_	325.0558	1.174	193
14	9.61	Caffeic acid	C_9_H_8_O_4_	179.0346	4.048	135
15	11.13	Kaempferol galactoside	C_21_H_20_O_11_	447.0920	−0.465	285
16	11.24	Quercetin-3-*O*-glucoside	C_21_H_20_O_12_	463.0869	−0.48	301
17	11.27	Syringic acid	C_9_H_10_O_5_	197.0452	3.655	182/153
18	11.34	*trans*-Resveratrol glucoside	C_20_H_22_O_8_	389.1233	0.504	227
19	11.72	Astilbin	C_21_H_22_O_11_	449.1078	−0.04	303
20	12.43	Quercetin-3-*O*-Rhamnoside	C_21_H_20_O_11_	447.0920	−0.465	301
21	12.55	*cis*-Resveratrol glucoside	C20H22O8	389.1233	0.504	227
22	12.65	*trans*-Resveratrol	C_14_H_12_O_3_	227.0706	4136	185
23	13.03	Kaempferol-3-*O*-glucoside	C_21_H_20_O_11_	447.0920	−0.465	285
24	16.8	Kaempferol	C_15_H_10_O_6_	285.0395	0.335	257
25	16.61	*cis-*Resveratrol	C_14_H_12_O_3_	227.0706	3080	185
26	16.84	dimetilcaffeic acid	C_11_H_12_O_4_	207.0657	2.486	179

## Data Availability

Not applicable.
